# Somatic Variant Analysis Identifies Targets for Tailored Therapies in Patients with Vascular Malformations

**DOI:** 10.3390/jcm9113387

**Published:** 2020-10-22

**Authors:** Stefano Paolacci, Raul Ettore Mattassi, Giuseppe Marceddu, Elena Manara, Alessandra Zulian, Giulia Guerri, Luca De Antoni, Carlo Arduino, Daniela Cavalca, Matteo Bertelli

**Affiliations:** 1 MAGI’S LAB, Via delle Maioliche, 57/D, 38068 Rovereto, TN, Italy; alessandra.zulian@assomagi.org (A.Z.); giulia.guerri@assomagi.org (G.G.); matteo.bertelli@assomagi.org (M.B.); 2 Center for Vascular Malformations “Stefan Belov”, Clinical Institute Humanitas “Mater Domini”, Via Gerenzano, 2, 21053 Castellanza, VA, Italy; raulmattassi@gmail.com; 3 MAGI EUREGIO, Via Maso della Pieve, 60/A, 39100 Bolzano, Italy; giuseppe.marceddu@assomagi.org (G.M.); elena.manara@assomagi.org (E.M.); luca.deantoni@assomagi.org (L.D.A.); 4 Medical Genetics Unit, City of Health and Science, Corso Bramante, 88, 10126 Turin, Italy; carloarduino@hotmail.com; 5 Laser Surgery Operating Unit, Plastic Surgery Department, San Rocco Clinical Institute, Via dei Sabbioni, 24, 25050 Ome, BS, Italy; daniela.cavalca2014@gmail.com; 6 EBTNA–LAB, Via delle Maioliche, 57/G, 38068 Rovereto, TN, Italy

**Keywords:** vascular malformations, next generation sequencing, tailored therapy

## Abstract

Vascular malformations include various disorders characterized by morphological, structural and/or functional alterations of blood and lymph vessels. Most are sporadic, due to somatic mutations. Here, we report a cohort of patients with sporadic and/or unifocal vascular malformations, in whom we carried out next generation sequencing analysis of a panel of genes associated with vascular malformations. The 115 patients analyzed were from different clinical centres. In 37 patients (32%), we found pathogenic mutations: most of these were gain–of–function mutations in *PIK3CA* (18%, 21/115) and *TEK* (13/115, 11%). We also found mutations in *GNAQ*, *CCM2* and *PTEN*. Identifying pathogenic variants in patients with vascular malformations can help improve management, particularly in cases with activating mutations that cause an increase in cell proliferation. Personalized pharmacological treatment, if possible, is now considered preferable to surgery and can help prevent recurrences, i.e., long–term complications of residual malformation or regrowth of tumors. For instance, rapamycin is currently being investigated for the treatment of various vascular malformations associated with hyperactivation of the phosphoinositide 3–kinase/Akt/mammalian target of rapamycin (PI3K/Akt/mTOR) pathway.

## 1. Introduction

Vascular malformations include various disorders characterized by morphological, structural and/or functional alterations of arteries, veins and/or capillaries. They affect roughly 0.3% of the population [1]. The classification universally used to define vascular anomalies is that adopted by the International Society for the Study of Vascular Anomalies (ISSVA) [2,3] (Table 1). It classifies vascular tumors and vascular malformations in the category of vascular anomaly. Vascular tumors usually have a growth cycle and some (e.g., hemangioma) regress later in life, whereas vascular malformations (e.g., arteriovenous malformations, arteriovenous fistula, capillary malformations, venous malformations and lymphatic malformations) are usually benign congenital lesions that may enlarge, worsen or become more visible/prominent over time. Malformations are further aggravated by blood or lymphatic pressure under physiological conditions. Fast–flow malformations include arterial malformations (AMs), arteriovenous malformations (AVMs) and arteriovenous fistulae (AVF); slow–flow lesions are capillary malformations (CMs), venous malformations (VMs), lymphatic malformations (LMs) and combined vascular malformations [4].

Autosomal dominant and recessive inheritance have been described for vascular malformations, although most have sporadic onset due to somatic mutations (typically only found in affected tissue or in mosaicism).

We screened a cohort of patients with sporadic and/or unifocal vascular malformations, using a panel of genes known to be associated with vascular malformations, in order to identify pathogenic somatic variants.

## 2. Materials and Methods

### 2.1. Patients

We retrospectively analyzed the results of genetic analysis of 115 anonymized patients from various clinical centres diagnosed with unifocal and/or sporadic vascular malformations. All subjects gave their informed consent for inclusion before they participated in the study, including authorization to use their genetic results for research and publication. The study was conducted in accordance with the Declaration of Helsinki, and the protocol was approved by the Ethics Committee of Azienda Sanitaria dell’Alto Adige, Italy (Approval No. 94–2016). All patients received genetic counseling to explain the risks and benefits of genetic testing.

### 2.2. DNA Extraction, Sequencing and Analysis

Genomic DNA from blood and biopsy samples was extracted by standard procedures using the MagPurix Blood or Tissue DNA Extraction Kit (Zinexts Life Science, New Taipei City, Taiwan).

A custom–made oligonucleotide probe library was designed to capture all coding exons and flanking exon/intron boundaries of 92 genes known or suspected to be associated with vascular disorders in humans and/or animal models. These genes were retrieved from the Human Gene Mutation Database (HGMD) professional, Online Mendelian Inheritance in Man (OMIM) database, Orphanet database, GeneReviews, PubMed and Mouse Genome Informatics (MGI). Sixteen of the 92 genes known to be affected by somatic mutations in patients with vascular malformations were included in the bioinformatic analysis (Table 1). The DNA probe set, complementary to the target regions (GRCh38/hg38), was designed using the specific online tool, Illumina DesignStudio (http://designstudio.illumina.com/Home/SelectAssay/), and was optimized to improve the coverage of low–performance target regions. In–solution target enrichment was performed according to the manufacturer’s protocol using the Nextera Rapid Capture Enrichment kit (Illumina). A MiSeq personal sequencer (Illumina) was used to perform 150 bp paired–end read sequencing according to the manufacturer’s instructions.

The raw data in fastq format, generated by the Illumina MiSeq on–instrument software, was analyzed to generate the final set of sequence variants using an in–house pipeline that includes the following modules: mapping, duplicate read removal, indel realignment, quality calibration, coverage analysis, variant calling and annotation. To detect somatic variants in vascular malformation biopsy specimens, we used a pipeline that exploits a subtractive correction method [5]. In order to isolate somatic variants with high confidence, we compared sequencing data obtained from biopsy and blood/saliva specimens and selected variants absent in blood/saliva, with at least 100 reads mapping in that region and with variant reads ≥5% with respect to wild–type reads.

The somatic variants identified were denoted as pathogenic or likely disease–causing if at least one of the following criteria was met: (1) the change was previously documented to be pathogenic, (2) the change results in a truncating mutation, (3) the change affects the canonical splice sites, (4) the change induces a start–or–stop loss, (5) the change has a deleterious effect predicted by at least two of three in silico pathogenicity prediction tools: Sorting Intolerant From Tolerant (SIFT, http://sift.jcvi.org/), Polymorphism Phenotyping version 2 (PolyPhen–2, http://genetics.bwh.harvard.edu/pph2/) and MutationTaster (http://mutationtaster.org/). All somatic variants identified after NGS were confirmed using the GenomeLab SNPStart Primer Extension Kit (BeckmanCoulter) according to the manufacturer’s protocol.

## 3. Results

### 3.1. Cohort Analysis

**Figure 1:**
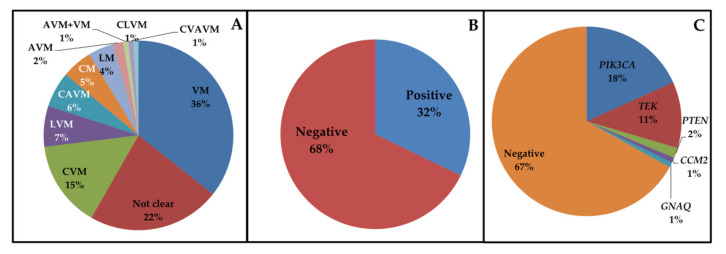
(**A**) Relative percentage of vascular malformations affecting patients. AVM = Arteriovenous malformation; CAVM = Capillary–Arteriovenous malformation; CLVM = Capillary–Lymphatic–Venous malformation; CM = Capillary malformation; CVAVM = Capillary–Venous–Arteriovenous malformation; CVM = Capillary–Venous malformation; LM = Lymphatic malformation; LVM = Lymphatic–Venous malformation; VM = Venous malformation. (**B**) Relative percentage of negative and positive cases after genetic testing. (**C**) Relative percentage of the mutated genes.

Sequencing of the patients (Figure 1A) produced a mean sequencing depth of the target region of 408× and 98% mean target coverage at 20×. Positive cases numbered 37/115 (32%) (Figure 1B). Most patients had gain–of–function mutations in functional domains of *PIK3CA* (18%, 21/115) or *TEK* (13/115, 11%) (Table 2, Figure 1C). In all cases, the variants were absent in DNA extracted from whole blood.

### 3.2. PIK3CA Mutations

**Figure 2:**
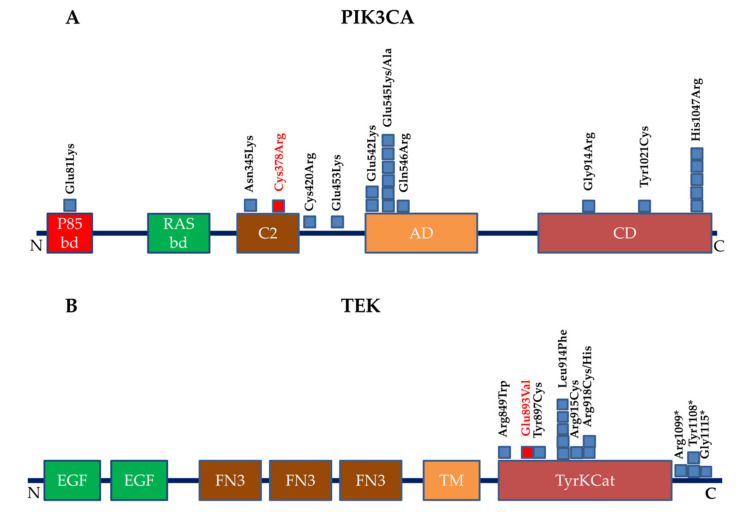
(**A**,**B**) schematic representation of the PIK3CA and TEK domains affected by the mutations that we found in this study (the squares indicate the variants found). The novel variants reported here are in red. p85bd = p85–binding domain; RASbd = Ras–binding domain; C2 = Protein kinase C conserved region 2; AD = Accessory domain; CD = catalytic domain; EGF = Epidermal growth factor–like domain; FN3 = Fibronectin type 3 domain; TM = Transmembrane domain; TyrKCat = Tyrosine kinase, catalytic domain.

We found pathogenic somatic mutations in *PIK3CA* (NM_006218.3) in 21 patients. Six out of 21 mutations (29%) affected the mutational hotspot Lys545 (Figure 2A). Although the same amino acid was mutated, the carriers had different phenotypes: one had lymphatic malformations, one lymphatic–venous malformations, one capillary–venous malformations and three had venous malformations. One patient carried a novel mutation, c.1132T > C; p.(Cys378Arg) associated with progressive venous phlebectasia, edema, pain and aplasia of lymphatic vessels of the right leg. This variant was considered pathogenic because it affects a residue that is already known to be mutated in *PIK3CA*–related disorders. Furthermore, this variant, according to MutationTaster, causes loss of the C2 domain. The same effect is predicted for the already reported variant in the same codon: c.1133G > A; p.C378Y. Since the C2 domain is essential for auto–inhibition of *PIK3CA*–encoded protein, this variant is predicted to be gain–of–function. Three had adipose hypertrophy near the vascular malformation and one patient with the mutation c.1357G > A; p.(Glu453Lys) showed varicocele and venous phlebectasia of the left leg, groin and knee with no signs of lymphatic deficit.

### 3.3. TEK Mutations

We found pathogenic somatic mutations in *TEK* (NM_000459.4) in 13 patients, 5/13 of whom (38%) showed mutations in the mutational hotspot Leu914 (Figure 2B). Eight patients had painful malformations. Two patients with p.(Leu914Phe) showed cutaneous capillary–venous malformations; this mutation was previously reported in cases with blue rubber bleb nevus syndrome. We also found a novel mutation, p.(Glu893Val), associated with a mucocutaneous venous malformation of the neck. This variant is located in the tyrosine kinase domain, like two other variants already found to be pathogenic: p.R849W and p.Y897C. The latter variants cause ligand–independent hyperphosphorylation of the TEK receptor [13,14]. We therefore assumed that our variant has a similar effect.

### 3.4. Other Mutations

In a biopsy from a patient who had a capillary–venous malformation near an eyebrow [20,21] we found a mutation in *GNAQ* (NM_002072.4), p.(Gln209Arg). As far as we know, this is the first time that this mutation has been linked to a capillary–venous malformation.

In another case, we found a novel mutation in *CCM2* (NM_31443.3), p.(Pro55Argfs*9) in a biopsy specimen from a subcutaneous venous lacuna in the right arm. Although this variant is new, we do not expect it to have a specific effect. Since this truncating variant is not located in the last exon, we expect it to trigger nonsense–mediated decay that would cause haploinsufficiency of *CCM2*. The patient also showed cerebral cavernous angioma, presumably linked to the same mutation. Unfortunately, a biopsy from that lesion was not available for analysis.

### 3.5. A Case with Both PTEN and TEK Pathogenic Variants

One patient had two somatic mutations in two different genes, *PTEN* (NM_000314.6), p.(Arg233*) and *TEK*, p.(Tyr1108*). Interestingly, the patient had two apparently unrelated malformations, an arteriovenous malformation in the supraclavicular region, removed surgically, and venous malformations in three parts of the body (leg, neck, abdomen). At 21 years of age, the patient sought medical attention for a mass in the right supraclavicular region (suspected to be an arteriovenous malformation), treated a year later with embolization and sclerotherapy. At 26 years of age, the mass was removed surgically. Seven years later, a mass appeared on the left scapula, diagnosed by MRI as a venous malformation, but the clinician decided not to treat it. At 35 years of age, the venous malformation was removed by phleboguided alcoholization. At the last examination, the patient showed intramuscular venous malformations of the left leg, neck and abdomen. This is a particular case in which venous malformations appeared several years after removal of an arteriovenous malformation. Since the two variants were both identified in the venous malformation of the neck, we cannot be absolutely sure that one variant was responsible for one phenotype and the other for the other. Unfortunately, we were only able to analyze the venous malformation biopsy material.

## 4. Discussion

Here, we again confirmed that most venous malformations are caused by somatic mutations in *PIK3CA* and *TEK* [22], although a genotype–phenotype correlation is difficult to establish, since patients with the same mutation can manifest different kinds of malformations. For instance, since most venous malformations affecting limbs are painful [23], we found such features in 8/10 patients with mutations in *TEK*, but in only 3/20 of patients with mutations in *PIK3CA*. However, genotype–phenotype correlations are not straightforward, as different patients with the same mutations may have different malformations. For instance, out of five patients with the mutation p.(Glu545Lys) in *PIK3CA*, two had an isolated venous malformation, one had a lymphatic malformation, one had a capillary–venous malformation, and another had a lymphatic–venous malformation. Conversely, different mutations can cause overlapping clinical conditions. This variability depends on where the mutation arises and at what stage of development.

It emerged from this study that the NGS gene panel tested must be broad. For instance, we found a novel mutation in *CCM2*, p.(Pro55Argfs*9), in a patient with subcutaneous venous lacunae and cerebral cavernous angioma: the latter can increase the risk of hemorrhage and/or seizure [24]. This finding implies that if a patient seeking medical attention for subcutaneous venous malformations is found to have a variation in a cerebral vascular malformation gene, then screening for endocranial vascular malformations is warranted.

We found a mutation in *GNAQ*, p.(Gln209Arg), previously reported as hyperactivating the RAS/MAPK pathway [5], in a patient with a capillary–venous malformation near the eyebrow. The p.Gln209Arg mutation has often been reported in uveal melanoma but never before in a case of capillary–venous malformation [5].

We also identified a patient with truncating mutations in *PTEN*, p.(R233*), and *TEK*, p.(Tyr1108*). The combination of these mutations, reported for the first time, may explain the concomitance of arteriovenous and venous malformations in that patient. Germline mutations in *PTEN* are known to cause complex disorders like Cowden syndrome, which entails various malformations not limited to blood vessels. In our case, a somatic loss–of–function mutation in *PTEN* caused an isolated arteriovenous malformation, clearly restricted to the site where the mutation occurred. The identification of the *PTEN* mutation can be useful not only for insights into the etiology of the malformations but also because *PTEN* mutations may be linked to the onset of various types of cancer [25]. On the other hand, the *TEK* mutation may explain the onset of venous malformations.

A limitation of our study is related to the threshold of 5% that we chose before we regarded a mutation as being responsible for a lesion. We thought it prudent to do so in order to be sure that calls of relevant somatic mutations were correct: however, if a biopsy did not contain a sufficient number of mutant cells, we might have missed a mutation whose abundance did not reach 5%. This could be one reason why our yield of mutations was only 32%. Another reason could be related to the heterogeneity of our cohort, which included patients with malformations that were difficult to classify. A third reason could be that the panel of candidate genes needs to be expanded further.

## 5. Conclusions

Identifying pathogenic mutations in patients with vascular malformations is not just an academic exercise. In fact, we found mostly activating mutations that cause an increase in cell proliferation, as in tumors. Treating the affected region with anticancer drugs may therefore be an adjuvant to surgery and may help prevent relapses after surgery [26]. A list of drugs that are being tested in vascular malformations is given in Table 3.

A good example is rapamycin, currently being tested for treatment of various vascular malformations associated with hyperactivation of the phosphoinositide 3–kinase/Akt/mammalian target of rapamycin (PI3K/Akt/mTOR) pathway [27] and linked to mutations in *PIK3CA* or *PTEN* [2]. Finding effective drugs to treat vascular lesions may open the way for surgical excision followed by personalized pharmacotherapy.

## Figures and Tables

**Table 1:** Genes analyzed with corresponding phenotypes.

Gene	Disease (ISSVA Classification)
*PIK3CA*	Common (cystic) LM, common VM, Klippel–Trenaunay syndrome, MCAP, CLOVES syndrome, CLAPO syndrome, FAVA syndrome
*TEK*	Common VM, familial VM cutaneo–mucosal, blue rubber bleb nevus syndrome
*GLMN*	Glomuvenous malformations
*MAP3K3*	Verrucous venous malformation
*KRIT1*, *CCM2*, *PDCD10*	Cerebral cavernous malformations
*GNAQ*	Congenital hemangioma, CM “Port–wine” stain, nonsyndromic CM, CM of Sturge–Weber syndrome
*GNA11*	Congenital hemangioma, CM with bone and/or soft tissue hyperplasia, diffuse CM with overgrowth
*RASA1*	CM–AVM, Parkes–Weber syndrome
*MAP2K1*	AVM, AVF
*AKT1*	Proteus syndrome
*GNA14*	Tufted angioma, pyogenic granuloma, KHE
*IDH1, IDH2*	Maffucci syndrome, spindle–cell hemangioma
*PTEN*	Bannayan–Riley–Ruvalcaba syndrome, hamartoma of soft tissue/angiomatosis of soft tissue

AVF, arteriovenous fistula; AVM, arteriovenous malformation; CM, capillary malformation; KHE, kaposiform hemangioendothelioma; LM, lymphatic malformation; VM, venous malformation; CLOVES, congenital lipomatous overgrowth, vascular malformations, epidermal nevi, skeletal/scoliosis and spinal abnormalities; CLAPO, lower lip CM + face and neck LM + asymmetry and partial/generalized overgrowth; FAVA, fibro adipose vascular anomaly; MCAP, megalencephaly–capillary malformation–polymicrogyria.

**Table 2:** Somatic pathogenic variants identified in positive cases, with allelic imbalance and clinical phenotype.

Gene	ID	Gender	Onset	Variant	rs	MAF (%)	Polyphen-2	SIFT	Mutation Taster	Allelic Imbalance	Malformation (Description, Site)	Other Features	Ref.
*PIK3CA*	Case 1	M	C	c.241G > A; p.E81K	rs1057519929	NR	PrD	D	DC	37%	LVM (Phlebectasia, lymphatic deficit in both legs)	OG (feet), NMD	[6]
	Case 2	F	I	c.1035T > A; p.N345K	rs121913284	NR	PrD	T	DC	8%	VM (Dilated subcutaneous veins, right leg)	Extra–, intramuscular AH	–
	Case 3	F	C	c.1132T > C; p.C378R	novel	NR	PrD	T	DC	6%	LVM (right leg)		–
	Case 4	F	I	c.1258T > C; p.C420R	rs121913272	NR	PrD	D	DC	7%	VM (Intramuscular, right upper limb)	–	[7]
	Case 5	M	U	c.1357G > A; p.E453K	rs1057519925	NR	PoD	T	DC	6%	VM (Diffuse superficial phlebectasias)		[8]
	Case 6	F	I	c.1624G > A; p.E542K	rs121913273	NR	PrD	D	DC	9%	LM (Intramuscular, left arm)	AH, MR	[9]
	Case 7	F	U	c.1624G > A; p.E542K	rs121913273	NR	PrD	D	DC	10%	CVM (Painful extramuscular, left arm)	–	[9]
	Case 8	F	U	c.1633G > A; p.E545K	rs104886003	0.0004%	PrD	D	DC	9%	VM (Subcutaneous intra– and extra muscular, hip and right leg)	–	[6]
	Case 9	F	U	c.1633G > A; p.E545K	rs104886003	0.0004%	PrD	D	DC	5%	VM (Right thigh and elbow)	–	[6]
	Case 10	F	C	c.1633G > A; p.E545K	rs104886003	0.0004%	PrD	D	DC	9%	CVM (Deep intramuscular, left arm)	–	[6]
	Case 11	F	C	c.1633G > A; p.E545K	rs104886003	0.0004%	PrD	D	DC	5%	LM (Painful, right arm)	–	[6]
	Case 12	M	C	c.1633G > A; p.E545K	rs104886003	0.0004%	PrD	D	DC	5%	LVM (Phlebectasia, lymphatic deficit, left limb)		[9]
	Case 13	M	I	c.1634A > C; p.E545A	rs121913274	NR	PrD	D	DC	5%	VM (Chest, left shoulder)	–	[10]
	Case 14	F	I	c.1637A > G; p.Q546R	rs397517201	NR	PrD	T	DC	7%	CVM (Left leg)	–	–
	Case 15	F	C	c.2740G > A; p.G914R	rs587776932	NR	PrD	D	DC	10%	VM (Right leg, shoulder)	dys	[6]
	Case 16	M	C	c.3062A > G; p.Y1021C	rs121913288	NR	PrD	D	DC	6%	LVM (Superficial, left leg)		[6]
	Case 17	M	U	c.3140A > G; p.H1047R	rs121913279	0.0004%	PoD	D	DC	8%	CVM (Painful, right hand)	–	[9]
	Case 18	M	U	c.3140A > G; p.H1047R	rs121913279	0.0004%	PoD	D	DC	8%	CVM (Intra– and extramuscular, right chest)	AH	[9]
	Case 19	F	I	c.3140A > G; p.H1047R	rs121913279	0.0004%	PoD	D	DC	6%	VM (Intramuscular, right leg)	–	[9]
	Case 20	F	I	c.3140A > G; p.H1047R	rs121913279	0.0004%	PoD	D	DC	5%	LVM (Subcutaneous, left leg)	–	[9]
	Case 21	F	C	c.3140A > G; p.H1047R	rs121913279	0.0004%	PoD	D	DC	>20%	CLVM	CLOVES	[9]
*TEK*	Case 22	F	C	c.[2545C > T; 2743C > T]; p.[R849W; R915C]	rs80338908; no rs	0.0004%; NR	PrD; PrD	D; D	DC; DC	6%;5%	VM (Finger)	–	[11,12]
	Case 23	F	I	c.2678A > T; p.E893V	novel	NR	PrD	D	DC	5%	VM (Mucocutaneous, neck)	–	–
	Case 24	M	I	c.[2690A > G; 2752C > T]; p.[Y897C; R918C]	rs80338909; no rs	NR; NR	PrD; PrD	D; D	DC; DC	6%; 5%	VM (Laterocervical)		[13,14]
	Case 25	F	I	c.2740C > T; p.L914F	no rs	NR	PrD	D	DC	10%	CVM (Painful extra– and intramuscular, shoulder and neck)		[15]
	Case 26	F	I	c.2740C > T; p.L914F	no rs	NR	PrD	D	DC	10%	VM (Painful intra– and extramuscular, right hip)	–	[15]
	Case 27	F	U	c.2740C > T; p.L914F	no rs	NR	PrD	D	DC	8%	VM (Painful, right arm)	AH	[15]
	Case 28	F	I	c.2740C > T; p.L914F	no rs	NR	PrD	D	DC	10%	CVM (Painful, right hand)	–	[15]
	Case 29	F	I	c.2740C > T; p.L914F	no rs	NR	PrD	D	DC	10%	VM (Painful, left arm and hand)	–	[15]
	Case 30	F	I	c.2753G > A; p.R918H	rs1554701458	NR	PrD	D	DC	6%	VM (Painful intra–and extramuscular, neck)	–	–
	Case 31	F	U	c.3295C > T; p.R1099*	no rs	NR	/	/	DC	9%	VM (Multiple subcutaneous, right arm)		[16]
	Case 32	M	C	c.3324T > A; p.Y1108*	no rs	NR	/	/	DC	8%	VM (Painful intramuscular, left thigh)	–	[16]
	Case 33	F	U	c.3343G > T; p.G1115*	no rs	NR	/	/	DC	9%	VM (Multiple painful, left hand, elbow, arm, axilla)	–	[17]
*PTEN*	Case 34	M	I	c.388C > T; p.R130*	rs121909224	0.001%	/	/	DC	5%	AVM + VM (AVM supraclavicular, VM intramuscular, leg, neck, abdomen)	–	[18]
*TEK*				c.3324T > A; p.Y1108*	no rs	NR	/	/	DC	8%			[16]
*GNAQ*	Case 35	F	U	c.626A > G; p.Q209R	rs121913492	NR	PrD	D	DC	12%	CVM (Eyebrow)		[19]
*CCM2*	Case 36	M	A	c.164_165del; p.P55Rfs*9	novel	NR	/	/	DC	6%	VM (Subcutaneous venous lacunae in right arm, cerebral cavernous angioma)	–	–
*PTEN*	Case 37	F	I	c.697C > T; p.R233*	rs121909219	NR	/	/	DC	15%	VM (Painful extra– and intramuscular, left knee)	–	–

KTS, Klippel–Trénaunay syndrome; LD, lymphatic deficit; OG, overgrowth; NMD, neuromotor delay; AH, adipose hypetrophy; CN, cutaneous nevi; dys, dysmetria; MR, muscle retraction; LM, lymphatic malformation; NR, not reported; PrD, probably damaging; PoD, possibly damaging; D, damaging; T, tolerated; DC, disease causing; MAF, minor allele frequency; VM, venous malformation; VMCM, cutaneo–mucosal VM; CLOVES, congenital lipomatous overgrowth, vascular malformations, epidermal nevi, skeletal abnormalities; C, congenital; I, infancy; A, adulthood; U, unknown; *PIK3CA*, isoform NM_006218.3; *TEK*, isoform NM_000459.4; *PTEN,* isoform NM_000314.6; *CCM2,* isoform NM_031443.3; *GNAQ*, isoform NM_002072.4. *, premature stop codon.

**Table 3:** Possible pharmacological treatments for vascular malformations.

Disease	Mutated Genes	Affected Pathways	Potential Drugs (ClinicalTrial.gov ID)
Venous malformation	*TEK*, *PIK3CA*	RAS/MAPK, PI3K/AKT	Rapamycin (NCT00975819, NCT03767660), regorafenib (NCT02736305), miransertib (NCT03317366)
Cerebral cavernous malformations	*KRIT1*, *CCM2*, *PDCD10*	PI3K/AKT, RHO/ROCK, RAS/MAPK	Propranolol (NCT03474614, NCT03523650, NCT03589014), simvastatin (NCT01764451), atorvastatin (NCT02603328)
Capillary malformations	*GNAQ*, *GNA11*	RAS/MAPK, PI3K/AKT	Rapamycin (NCT00800722), hemoporfin (NCT03181984), bosentan (NCT02317679)
Arteriovenous malformations (sporadic, in HHT, in CM–AVM) and arteriovenous fistula	*GDF2, MAP2K1*, *ENG, ACVRL1, SMAD4*, *RASA1*, *EPHB4*	PI3K/AKT, TGF–β/BMP, RAS/MAPK	Tamoxifen (NCT00375622), doxycycline hyclate (NCT03397004), tranexamic acid (NCT01031992), marimastat (NCT00261391), rapamycin (NCT02042326), bevacizumab (NCT02314377, NCT01402531, NCT01397695), minocycline/doxycycline (NCT00243893), thalidomide (NCT00389935, NCT00964496), doxycycline (NCT00783523), atorvastatin (NCT03188978)
Lymphatic malformations	*PIK3CA*, *FLT4*, *VEGFC, GJC2*, *FOXC2*, *SOX18*, *GATA2*, *CCBE1*, *KIF11*, *PTPN14*	RAS/MAPK, PI3K/AKT, VEGF/VEGFR	Sildenafil (NCT01290484), picibanil (NCT03427619), rapamycin (NCT04128722), alpelisib (NCT03941782), marimastat (NCT00261391)

AVM, arteriovenous malformation; CM, capillary malformation; HHT, hereditary hemorrhagic telangiectasia.
